# Draft genome sequence of two *Fusobacterium varium* strains isolated from patients in Kazakhstan with colorectal cancer

**DOI:** 10.1128/mra.00205-24

**Published:** 2024-08-20

**Authors:** Botakoz Kurentay, Alua Gusmaulemova, Talgat Utupov, Dana Auganova, Pavel Tarlykov, Asset Daniyarov, Saule Khamzina, Meiram Mamlin, Arman Kozhakhmetov, Sanzhar Shalekenov, Gulmira Kulmambetova

**Affiliations:** 1 National Center for Biotechnology, Astana, Kazakhstan; 2 National Laboratory Astana, Nazarbayev University, Astana, Kazakhstan; 3 L.N. Gumilyov Eurasian National University, Astana, Kazakhstan; 4 National Research Oncology Center, Astana, Kazakhstan; 5 Nazarbayev University School of Medicine, Astana, Kazakhstan; University of Maryland School of Medicine, Baltimore, Maryland, USA

**Keywords:** *Fusobacterium varium*, genome sequence, colorectal cancer

## Abstract

*Fusobacterium varium* is a Gram-negative, invasive, obligate anaerobe in the gastrointestinal tract microbiota, associated with various clinical conditions, including colorectal cancer (CRC). Here, we announce the draft genome sequence of two *F. varium* strains Fv36kaz and Fv63kaz from patients with CRC in Kazakhstan.

## ANNOUNCEMENT

*Fusobacterium varium*, an opportunistic pathogen, is mostly reported in ulcerative colitis and should be considered a potential cause of colorectal cancer (CRC) (1–4).

Biopsy samples were collected from the CRC tissue of patients from the National Research Oncology Center. The homogenized solution from the biopsy, which was stored in 20% sucrose, was cultivated on Columbia agar (5% defibrinated sheep blood, 1 µg/mL menadione, 5 µg/mL hemin, 3 µg/mL josamycin, 4 µg/mL vancomycin, and 1 µg/mL neomycin) and incubated for 72 h under anaerobic conditions in an AnaeroGen system (Thermo) at 37°C. The isolated single colonies were identified as *F. varium* by V1–V4 region fragment of *16S rRNA* gene sequencing with more than 99% similarity for strains and the Bruker Biotyper microbial identification system. About 20 µL reactions mix contained template DNA, 0.2 U Taq Polymerase, 2 mM each dNTP, 10× PCR buffer, 25 mM MgCl_2_, and 10 pmol primers (8 f 5′-AGAGTTTGATCCTGGCTCAG-3′, 806R 5′-GGACTACCAGGGTATCTAAT-3′). The PCR annealing temperature was set to 55°C for 30 cycles. Sequencing was performed using a 3730 Genetic Analyzer (Thermo). The comparative method was constructed using the NCBI BLAST nucleotide, comparing the sequencing of both strains to the reference *Fv* (ATCC 27725). For DNA isolation, the colonies were grown at 37°C in Columbia agar (5% defibrinated sheep blood, 1 µg/mL menadione, 5 µg/mL hemin, 3 µg/mL josamycin, 4 µg/mL vancomycin, and 1 µg/mL neomycin) for 72 h under anaerobic condition. DNA was extracted using a QIAamp DNA mini kit (Qiagen). An Illumina MiSeq platform was used to carry out whole-genome sequencing, and a MiSeq reagent v3 600 cycles cartridge was used. DNA libraries were prepared using an Illumina DNA Prep kit. The total number of reads for Fv36kaz was 3,082,394, and the total number of reads for Fv63kaz was 2,402,302. Quality control and trimming of forward and reverse reads for both strains Fv36kaz and Fv63kaz were performed using Trim Galore version 0.6.5dev (5). The genome was assembled using Unicycler v0.4.8 (6). Contigs were annotated using the NCBI Prokaryotic Genome Annotation Pipeline (PGAP) v6.6 (7). Default parameters were used for all software. The genome of *F. varium* strain Fv36kaz with 72-fold coverage had a length of 3,388,962 bp, 145 contigs, an *N*_50_ value was 74,423 bp, and a G + C content of 29.01%. The genome of *F. varium* strain Fv63kaz with 185-fold coverage had a length of 3,393,562 bp, 107 contigs, an *N*_50_ value was 91,866 bp, and a G + C content of 29.11%. Additionally, annotation by the Bacterial and Viral Bioinformatics Resource Center Annotation Service of the Fv36kaz had 3,262 coding DNA sequences (CDSs), and Fv63kaz had 3,221 CDSs (8).

## Data Availability

This Whole Genome Shotgun project has been deposited at DDBJ/ENA/GenBank under the accession JBAJGW000000000 (Fv36kaz) and JBAKJD000000000 (Fv63kaz). The raw data from BioProject numbers PRJNA1078108 (Fv36kaz) and PRJNA1079513 (Fv63kaz) were submitted to the NCBI Sequence Read Archive under experiment accession numbers SRR28022417 (Fv36kaz) and SRR28059462 (Fv63kaz). The *16S rRNA* sequences from two *F. varium* were deposited in GenBank as separate submissions under accession numbers PP789727-PP789728 (Fv36kaz) and PP800236-PP800237 (Fv63kaz).
